# Mitigating risks in central line-associated bloodstream infection: a comprehensive insight into critical care nurses’ knowledge, attitudes, barriers, and compliance

**DOI:** 10.1186/s12912-024-02168-5

**Published:** 2024-07-20

**Authors:** Talal Ali Hussein Alqalah

**Affiliations:** 1 Department of Medical Surgical Nursing, College of Nursing, University of Ha’il, Ha’il City, Saudi Arabia; 2 Nursing Division, Faculty of Medicine and Health Sciences, Amran University, Amran, Yemen

**Keywords:** CLABSI prevention, Critical care nurses, Knowledge, Attitude, Barriers, Compliance, Multilayer neural network

## Abstract

**Background:**

Central venous catheter-related bloodstream infections (CLABSIs) are a significant concern in intensive care units (ICUs) as they lead to increased morbidity, mortality, and healthcare costs. Fortunately, these infections are largely preventable through strict adherence to CLABSI prevention guidelines. Nurses play a critical role in preventing CLABSIs.

**Aim:**

This study aimed to investigate factors affecting critical care nurses' knowledge, attitudes, and perceived barriers related to implementing CLABSI prevention guidelines, and to predict factors influencing compliance with these guidelines.

**Methods:**

This cross-sectional study was conducted from April to May 30, 2023, with a convenience sample of 470 critical care nurses from ICUs across eight hospitals in Sana’a, Yemen. Data were collected using an observational checklist and self-administered questionnaire. Descriptive statistics, Independent Student’s t-test, one-way ANOVA, Pearson’s correlation coefficient, multiple linear regression, and multilayer perceptron neural networks were performed.

**Results:**

Critical care nurses exhibited low knowledge of CLABSI prevention guidelines, with compliance reaching an acceptable level. Despite the higher perceived barriers, the nurses demonstrated a positive attitude. Nurses with greater knowledge and positive attitudes displayed higher compliance levels. However, perceived barriers were negatively associated with knowledge and compliance. Notably, multilayer neural network analysis identified knowledge and perceived barriers as the strongest predictors of nurses' compliance.

**Conclusion:**

The current findings emphasize the need for multifaceted strategies to implement the CLABSI prevention guidelines. These strategies should address knowledge gaps, support positive attitudes, and address practical barriers faced by nurses to ensure successful implementation of CLABSI prevention.

**Supplementary Information:**

The online version contains supplementary material available at 10.1186/s12912-024-02168-5.

## Introduction

Central venous catheters (CVCs) are widely used in intensive care units and considered safe for medication administration. Despite its benefits, CVCs are associated with CLABSI, which is the most common nosocomial infection, accounting for 20–30% of all hospital-acquired infections. Notably, CLABSI rates differ significantly between developed 1.2 and developing countries 6.5 per 1000 catheters [1].

Numerous studies have identified various risk factors of CLABSIs. These factors, as highlighted in both systematic review studies and meta-analyses, include total parenteral nutrition, chemotherapy, immunosuppression, and duration of catheterization [2]. However, it is crucial to underscore the practices of health-care workers, particularly nurses, regarding CVCs. Given their significant role in managing and maintaining CVCs, these practices can greatly influence CLABSIs. Consequently, a lack of knowledge and compliance with infection control protocols, coupled with the existence of barriers to effective practice, can contribute to an increased risk of CLABSIs [3].

CLABSIs are a significant source of morbidity, mortality, and economic burden in the healthcare system. Fortunately, these infections can be prevented by implementing rigorous aseptic techniques, robust surveillance programs, and evidence-based management guidelines [4]. The Centers for Disease Control and Prevention serve as a valuable resource, providing guidelines and tools to healthcare professionals to eliminate CLABSIs [5].

Nurses may not consistently comply with evidence-based guidelines for the prevention of CLABSIs because of a combination of internal and external barriers. Internal barriers can encompass attitudes and knowledge as well as cultural aspects and motivation. On the other hand, external barriers may include the design of the guidelines, resource availability, and the quality of leadership. Specifically, guidelines that are unclear or inflexible, lack the necessary resources, and ineffective leadership can all contribute to non-compliance [6].

Given the scarcity of local and regional research on this topic, this study was designed to investigate the factors affecting Yemeni critical care nurses’ knowledge, attitudes, and barriers related to implementing CLABSI prevention guidelines, and to predict factors affecting compliance with these guidelines.

## Methods

### Study design and setting

This cross-sectional study was conducted in the intensive care units of major teaching, public, and private hospitals in Yemen from April to May 30, 2023.

### Participants and sample

The study was conducted across 16 ICUs encompassing a range of specializations (trauma, surgery, coronary care, medical, neurology, burns, pediatrics, and post-cardiac surgery) within eight diverse hospitals (public, teaching, and private) in Sana'a, Yemen's most populous city. A convenience sampling approach was adopted to recruit 470 critical care nurses, ensuring an adequate sample size for multiple linear regression analysis (generally, one independent variable per 10 participants [7]). Eligible participants were registered ICU nurses who provided written informed consent on the first page of the questionnaire. Nurses on leave during the data collection period or with less than six months of ICU experience were excluded.

## Measures

Data were collected from critical care nurses working in intensive care units (ICUs) using a structured observational checklist and a self-administered questionnaire. The questionnaire was adapted and modified based on a review of relevant literature from similar studies [8–12]. The questionnaire was adopted in English version.

### Demographic characteristics

Demographic characteristics of critical care nurses, such as age, sex, education level, marital status, ICU experience, nurses-to-patient ratio, previous CLABSI prevention training, availability of a CLABSI prevention protocol, and type of hospital, were gathered.

### knowledge

The critical care nurses ' Knowledge Questionnaire on CLABSI Prevention was adapted from a questionnaire designed by Labeau et al. [8]. It consisted of 11 multiple-choice questions, each with one correct and three incorrect options. These questions covered various aspects of catheter care, including replacement timing, disinfection methods, set replacement, dressing of the catheter area, and methods for evaluating infection at the catheter site [8]. Each correct response received one point, whereas the incorrect response scored zero.

### Attitudes

Critical care nurses' attitudes toward evidence-based recommendations for preventing CLABSI were assessed using an 8-item questionnaire designed by Bianco et al. [9]. Each item was rated on a 3-point Likert scale, with a higher score indicating a more positive attitude.

### Barriers

This tool was adapted from the studies by Chen et al. [10] and Badparva et al. [11] to identify the barriers faced by critical care nurses in implementing evidence-based guidelines for CLABSI prevention. It consists of six barriers, with each item scored on a scale of 1–10. Higher scores indicated greater perceived barriers.

### Compliance

A structured observational sheet, originally derived from the Aloush and Alsaraireh study [12], was primarily designed to monitor CVC maintenance care. It assessed compliance with 10 CLABSI prevention guidelines using a three-point scale: 2 points for "done completely," 1 point for "done incompletely," and 0 points for "not done." [12]. The highest score indicated greater compliance among the critical care nurses.

### The validity and reliability of the instrument

The validity of all instruments was assessed by a panel of 14 experts, including members of five critical care nursing faculties, three infection control specialists, three ICU nurses, and three ICU clinical instructors. The final version of the questionnaire achieved content validity ratios and indices above 0.8, indicating its approval. In a pilot study involving 40 critical care nurses, the instruments demonstrated high reliability, with intraclass correlation coefficients of 0.89, 0.82, 0.79, and 0.84 for knowledge, barriers, attitude, and compliance, respectively.

### Data collection

This study was approved by the Research Ethical Committee of Alrazi University (56/FMS/2021). The data collection involved direct observation and self-administered questionnaires. Initially, critical care nurses were informed that observation would occur; however, the purpose of the study and specific aspects of their work to be observed were not disclosed. Data on the compliance of critical care nurses with CLABSI prevention guidelines in ICUs were collected through unobtrusive direct observation during all three shifts. These observations were conducted by trained nurses in each ICU who had received educational sessions on the best evidence-based practices for CLABSI prevention and how to use a structured observational checklist. Once the observation was completed, the critical care nurses were informed that they had been observed during CVC care. They were given the right to participate after the purpose of the study was explained. A self-administered questionnaire assessing knowledge, attitudes, and barriers was distributed. The structured observation checklist was coded congruently with the self-administered questionnaire to ensure that the data for each nurse could be matched easily. Data were collected anonymously to ensure participant confidentiality.

## Statistical analysis

Trained nurses collected questionnaires from the participants, checked for missing data, and returned incomplete questionnaires to the participants for completion. Subsequently, the collected data were encoded and analyzed using the Statistical Package for the Social Sciences version 27. All items within the dimensions of knowledge, attitudes, perceived barriers, and compliance were summed to obtain a total score. This score was then assessed for normality using a histogram and statistical tests (Kolmogorov–Smirnov and Shapiro–Wilk tests). Since the *p*-values of these tests were greater than .05, the data were considered normally distributed. The relationships between nurses' characteristics and the total score were explored using independent-sample t-tests (for two groups) and one-way ANOVA (for more than two groups). Pearson's correlation coefficients were used to identify correlations between dimensions. Additionally, multiple linear regression analysis was conducted to determine the factors that predicted critical care nurses' knowledge, attitudes, and perceived barriers. There was no evidence of substantial multicollinearity, as indicated by a variance inflation factor that did not exceed four. The explanatory power of the model was assessed using R-squared statistics.

Nurses’ compliance scores were transformed into binary variables. Scores below 10 were categorized as “insufficient compliance,” while scores of 10 or higher were deemed as “sufficient compliance” [12]. This new binary variable of the nurses’ scores served as the dependent variable in the multilayer neural network. A multilayer perceptron neural network was employed to predict critical care nurses' compliance with the CLABSI prevention guidelines. The network considered features including knowledge, attitudes, barriers, demographics (age, sex, marital status, educational level, and experience), and hospital factors (workload ratio, hospital type, availability of CLABSI prevention protocol, and training course participation). The network architecture consisted of an input layer with 27 neurons representing the pre-processed input feature. This was followed by one hidden layer with six neurons and a final output layer with two neurons to classify the nurses' compliance as insufficient or sufficient (binary classification).

## Results

### Critical care nurses characteristics and association with knowledge, attitudes, perceived barriers, and compliance

The total number of observed critical care nurses was 500, 470 of whom agreed to complete a self-administered questionnaire. The overall response rate was 94%. The mean age was 33.7 years; 33.5 years for females and 33.8 years for males. Of the participants, 61.5% were aged between 31 and 40 years, 52.6% were females, and 59.8% had a bachelor’s degree in nursing science. More than half (56.8%) were married, and 44.0% had less than 5 years of experience. More than two-thirds (65.7%) had no previous training in CLABSI prevention. The majority (79.6%) reported that no CLABSI prevention protocol was available at their hospital. Of these, 43.2% took care of three patients per shift.

Critical care nurses older than 40 years had higher mean scores for knowledge, compliance, and attitudes and a lower mean score for perceived barriers. The difference in the knowledge scores was statistically significant (*p* = .004). Nurses with a master’s degree or those who have undergone prevention training or have a prevention protocol in their hospitals have significantly higher mean scores for knowledge, attitudes, and compliance and lower mean scores for perceived barriers, where the *p*-value was less than .05. Nurses with more than 10 years of experience and those caring for one patient per shift had the highest mean scores for knowledge and compliance, and lower mean scores for perceived barriers, with *p*-values less than .05. Nurses working in teaching hospitals had the highest mean scores for knowledge, compliance, and attitudes, with *p*-values of .023, .027 and .019 respectively. However, the scores for the perceived barriers were not significantly different. The findings are summarized in Table 1.

**Table 1:** Critical care nurses characteristics and association with knowledge, compliance, attitudes, and barriers (*N* = 470)

Variable		Overall F(%)	Knowledge Mean ± SD	Attitude Mean ± SD	Barriers Mean ± SD	Compliance Mean ± SD
**Age **^**(b)**^	≤ 30 years	123(26.2)	20.59 ± 2.97	39.40 ± 9.23	5.00 ± 1.67	12.09 ± 3.41
	31 to 40 years	289(61.5)	21.35 ± 3.04	38.45 ± 9.05	5.44 ± 1.58	12.62 ± 3.30
	> 40 years	58(12.3)	21.06 ± 3.12	36.57 ± 9.15	5.84 ± 2.04	13.10 ± 3.98
	***p*-value**		.**004**	.082	.202	.151
**Sex **^**(a)**^	Male	213(47.4)	20.92 ± 3.32	38.64 ± 9.01	5.27 ± 1.70	12.51 ± 3.39
	Female	247(52.6)	21.29 ± 3.06	38.31 ± 9.93	5.47 ± 1.67	12.57 ± 3.46
	***p*-value**		.210	.208	.724	.862
**Educational level **^**(b)**^	Diploma	138(29.3)	20.57 ± 3.58	40.73 ± 8.64	4.99 ± 1.65	11.75 ± 3.22
	Bachelor	281(59.8)	21.28 ± 3.02	37.51 ± 8.13	5.46 ± 1.63	12.81 ± 3.45
	Master	51(10.9)	21.68 ± 2.76	37.62 ± 8.46	5.94 ± 1.90	13.21 ± 3.52
	***p*-value**		** < .001**	**.041**	**.006**	**.004**
**Marital status **^**(b)**^	Single	176(37.5)	21.06 ± 3.32	38.36 ± 9.58	5.43 ± 1.75	12.48 ± 3.55
	Married	267(56.8)	21.14 ± 3.13	38.63 ± 9.37	5.35 ± 1.61	12.54 ± 3.33
	Divorce/Widow	27(5.7)	21.33 ± 2.78	37.51 ± 9.68	5.25 ± 1.90	12.92 ± 3.52
	***p*-value**		.843	.909	.846	.823
**Experience **^**(b)**^	≤ 5 ears	207(44.0)	21.11 ± 2.81	39.71 ± 8.06	5.21 ± 1.50	12.25 ± 3.03
	6 to10 years	145(30.9)	21.05 ± 3.31	39.39 ± 9.39	5.34 ± 1.68	12.33 ± 3.46
	> 10 years	118(25.1)	21.22 ± 3.06	36.37 ± 9.96	5.71 ± 1.94	13.32 ± 3.90
	***p*-value**		**.037**	.906	**.014**	**.017**
**Previous CLABSI prevention training **^**(a)**^	Yes	161(34.3)	21.51 ± 2.97	37.21 ± 9.40	5.60 ± 1.72	13.14 ± 3.45
	No	309(65.7)	20.91 ± 3.27	39.12 ± 9.67	5.26 ± 1.65	12.23 ± 3.37
	***p*-value**		**.020**	**.027**	**.024**	**.003**
**Availability of CLABSI prevention protocol **^**(a)**^	Yes	96(20.4)	21.62 ± 2.48	36.01 ± 9.21	5.73 ± 1.76	13.25 ± 3.69
	No	370(79.6)	20.99 ± 3.33	39.10 ± 9.03	5.28 ± 1.65	12.31 ± 3.31
	***p*-value**		**.019**	**.041**	**.022**	**.006**
**Type of hospital **^**(b)**^	Public	147(31.3)	20.61 ± 3.84	38.82 ± 9.03	5.14 ± 1.68	12.16 ± 3.66
	Private	161(34.3)	21.08 ± 3.02	39.21 ± 9.13	5.31 ± 1.70	12.31 ± 3.34
	Teaching	162(34.4)	21.62 ± 2.56	37.41 ± 9.69	5.66 ± 1.64	13.12 ± 3.22
	***p*-value**		**.023**	**.019**	.235	**.027**
**Nurses to patients’ ratios **^**(b)**^	1:1	89(18.9)	21.49 ± 3.32	36.85 ± 9.88	5.83 ± 1.95	13.15 ± 3.83
	1:2	178(37.9)	21.25 ± 3.17	37.79 ± 9.41	5.50 ± 1.76	12.73 ± 3.51
	1:3	203(43.2)	20.84 ± 3.12	39.77 ± 8.97	5.07 ± 1.41	12.11 ± 3.11
	***p*-value**		** < .001**	.212	**.036**	**.037**

^(a)^ Student’s t-test

^(b)^ One-way ANOVA

SD Standard deviation

### Critical care nurses’ attitudes toward evidence-based recommendations for preventing CLABSI

Critical care nurses had positive attitudes toward the general aspects of CLABSI prevention (21.12 out of 24). They believe that maintaining aseptic techniques for the insertion and care of CVC reduces the CLABSI risk. They also agreed that antiseptics should be allowed to dry before catheter placement and hand hygiene should be performed before and after CVC care. Agreement rates for these practices were 88.7%, 83.4%, and 80.9%, respectively (Table 2).

**Table 2:** Critical care nurses attitudes toward Attitudes toward evidence-based recommendations for preventing CLABSI (*N* = 470)

Attitudes	Agree	Uncertain	Disagree
Maintaining aseptic technique for the insertion and care of intravascular catheters reduces CLABSI risk	88.7^a^	6.2	5.1
Performing hand hygiene before and after inserting, replacing, accessing, or dressing an intravascular catheter reduces CLABSI risk	80.9^a^	8.3	10.9
If patients have fever without obvious source, catheter site dressing should be removed to allow thorough examination of the site	72.6^a^	14.0	13.4
Catheter insertion site should be monitored visually or by palpation through an intact dressing on a regular basis	55.7^a^	19.6	24.7
Using topical antibiotic ointment or creams on insertion CVC sites reduces CLABSI risk	13.6	16.0	70.4^a^
Antiseptics should be allowed to dry prior to placing the catheter	83.4^a^	9.1	7.4
Routine CVCs replacement is effective to prevent CLABSI	12.3	12.2	75.5^a^
Using a CVC with the minimum number of lumens is an effective practice to reduce CLABSI risk	79.8^a^	13.0	7.2

^a^ correct answer

### Perceived barriers to implementation of CLABSI prevention guidelines in ICU

As illustrated in Table 3, critical care nurses identified several higher barriers to implementing evidence-based CLABSI prevention guidelines. These included overwhelming workload (mean score: 7.05), shortage of nursing staff (mean score: 6.78), lack of CLABSI prevention workshops (mean score: 6.66), and shortage of necessary equipment (mean score: 6.42).

**Table 3:** Perceived barriers to implementation of CLABSI prevention guidelines in ICU (*N* = 470)

Barriers	Rate										Mean ± SD
	**1**	**2**	**3**	**4**	**5**	**6**	**7**	**8**	**9**	**10**	
Unfamiliar with the guidelines	27(5.7)	26(5.5)	14(3)	59(12.6)	41(8.7)	46(9.8)	63(13.4)	144(30.6)	44(9.4)	6(1.3)	6.14 ± 2.31
Lack of CLABSI prevention workshops/training	10(2.1)	19(4)	19(4)	46(9.8)	43(9.1)	52(11.1)	46(9.8)	173(36.8)	26(5.5)	36(7.7)	6.63 ± 2.22
A nurse shortage	15(3.2)	11(2.3)	24(5.1)	33(7)	38(8.1)	44(9.4)	51(10.9)	181(38.5)	42(8.9)	31(6.6)	6.78 ± 2.21
High workload	10(2.1)	18(3.8)	16(3.4)	33(7)	22(4.7)	49(10.4)	67(14.3)	140(29.8)	55(11.7)	60(12.8)	7.05 ± 2.26
Shortage of necessary equipment	33(7)	28(6)	32(6.8)	33(7)	29(6.2)	34(7.2)	45(9.6)	124(26.4)	77(16.4)	35(7.4)	6.42 ± 2.70
Lack of rewards and punishments policy	22(4.7)	42(8.9)	45(9.6)	48(10.2)	101(21.5)	60(12.8)	49(10.4)	36(7.7)	39(8.3)	28(6)	5.45 ± 2.42

### Correlation matrix association between nurses knowledge, attitudes, barriers, and compliance.

This study revealed a robust link between critical care nurses' knowledge and clinical behaviors. Notably, higher knowledge scores demonstrated a strong positive association with both compliance (*r* = 0.781) and positive attitudes (*r* = 0.868) towards CLABSI prevention. Interestingly, the positive influence extended further, with improved attitudes fostering greater compliance (*r* = 0.784). The findings also revealed a significant negative association between increased knowledge, compliance, positive attitudes, and perceived barriers to CLABSI prevention guideline implementation (*r* = -0.639, -0.861, and -0.610 respectively), as shown in Table 4.

**Table 4:** Correlation matrix between critical care nurses’ knowledge, compliance, attitudes, and barriers (*n* = 470)

		**Knowledge (5.3 ± 1.6)**	**Compliance (12.5 ± 3.4)**	**Attitude (21.1 ± 3.1)**	**Barriers (38.4 ± 9.9)**
**Knowledge**	Pearson Correlation	1			
	Sig. (2-tailed)				
**Compliance**	Pearson Correlation	.781^b^	1		
	Sig. (2-tailed)	< .001			
**Attitude**	Pearson Correlation	.868^b^	.784^b^	1	
	Sig. (2-tailed)	.000	.000		
**Barriers**	Pearson Correlation	-.639^b^	-.861^b^	-.610^b^	1
	Sig. (2-tailed)	< .001	< .001	< .001	

^b^. Correlation is significant at the 0.01 level (2-tailed)

### Prediction of critical care nurses knowledge, compliance, attitudes, and barriers

Multiple linear logistic regression models were used with the following four dependent variables: Knowledge, compliance, attitudes, and barriers. The models explained 80% of the variance in nurses’ knowledge, 9% in compliance, 62% in attitude, and 76% in perceived barriers.

Nurses aged less than 30 years and those from 31 to 40 years old had knowledge scores that were 0.49 and 0.44 points lower, respectively, than those aged over 40 years. Nurses with a diploma degree had knowledge scores that were 0.36 points lower than those with a master’s degree. Meanwhile, nurses who cared for ICU patients at a ratio of 1:1 and 1:2 had knowledge scores that were 0.43 and 0.19 points higher, respectively, than those who cared for three ICU patients. Higher knowledge was associated with 0.11- and 0.36-point increases in nurses’ compliance and attitudes, respectively, compared to those with low knowledge.

Critical care nurses with less than five years of experience had compliance scores that were 0.86 points lower than those with more than 10 years of experience in the ICU. Nurses who worked in hospitals where a CLABSI protocol was available had compliance scores that were.96 points higher than those who worked in hospitals without a CLABSI protocol. On the other hand, nurses who worked in public and private hospitals had compliance scores that were 0.76 and 0.91 points lower, respectively, than those working in teaching hospitals. Those who cared for one patient had compliance scores that were 1.02 points higher than those who cared for three patients in the ICU. Nurses with higher positive attitudes had compliance scores that were 0.73 points higher than those with lower attitudes.

Moreover, lower compliance was associated with higher barriers faced by the nurses. Nurses who faced barriers to the implementation of evidence-based guidelines for CLABSI prevention had compliance scores that were 2.88 points lower than those who did not face such barriers, as shown in Table 5.

**Table 5:** Multiple linear regression models estimating the effect of the nurses characteristics on knowledge, compliance, attitudes, and barriers scores (*N* = 470)

Variable	Knowledge			Attitudes			Barriers		
	OR	*p*-value	95% CI for odds ratio	OR	*p*-value	95% CI for odds ratio	OR	*p*-value	95% CI for odds ratio
**Age**									
≤ 30 years	-.49	** < .001**	-.77–.20						
31 to 40 years	-.44	** < .001**	-.68–.20						
> 40 years	reference								
**Educational level**									
Diploma	-.36	**.005**	-.61–.11	-.02	.944	-.67-.62	-.47	.566	-2.09–1.15
Bachelor	-.21	.072	-.44-.02	-.10	.749	-.69-.49	-1.14	.133	-2.63–0.35
Master	reference								
**Experience**									
≤ 5 ears	-.13	.221	-.34-.08				.37	.534	-.79–1.51
6 to10 years	-.03	.742	-.23-.17				-.75	.230	-1.10-.48
> 10 years	reference								
**Training about prevention of CLABSI**									
Yes	-.08	.307	-.23-.07	-.07	.741	-.45-.32	.50	.311	-.47–1.47
No	reference								
**Availability of CLABSI protocol**									
Yes	.07	.465	-.11-.24	-.23	.303	-.69-.22	-.23	.695	-1.36-.91
No	reference								
**Type of hospital**									
Public	-.02	.860	-.16-.19	-.33	.145	-.85–.01			
Private	-.07	.403	-.24-.10	.06	.793	-.48-.34			
Teaching	reference								
**Nurses to patients’ ratios**									
1:1	.43	** < .001**	.23-.62				-.35	.589	-1.60-.91
1:2	.19	**.017**	.03-.34				-.38	.461	-1.39-.63
1:3	reference								
**Compliance**	.11	** < .001**	.07-.14	.73	** < .001**	.68-.79	-2.88	** < .001**	-3.09–2.67
**Attitude**	.36	** < .001**	.33-.40				-.52	** < .001**	-.29–.38

Knowledge model: R Square = .80, Adjusted R Square = .79, F = 132.51, *p* < .001

Attitudes model: R Square = .62, Adjusted R Square = .61, F = 107.17, *p* < .001

Barriers model: R Square = .76, Adjusted R Square = .75, F = 143.23, *p* < .001

**Fig. 1:**
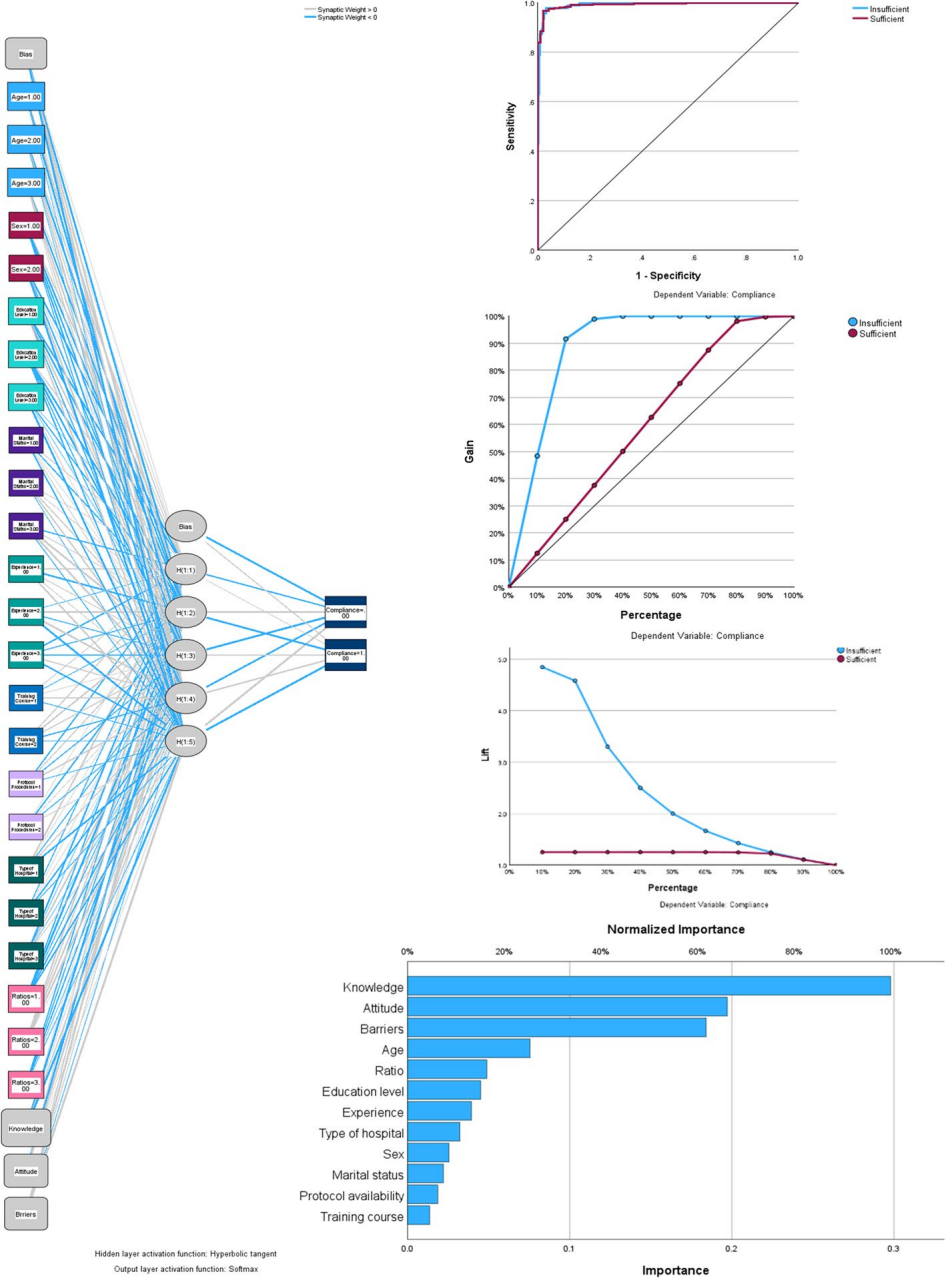
Multilayer neural network for predicting compliance of critical care nurses to CLABSI prevention guidelines

The implemented multilayer neural network achieved a high accuracy in predicting nurse complaints based on the provided features, including knowledge, attitudes, barriers, demographics, and hospital factors. The training accuracy reached 96.3% and the testing accuracy was even higher (96.6%). Additionally, the area under the ROC curve for both insufficient and sufficient compliance was exceptional at 0.993, indicating the model's strong performance in classifying compliance. Interestingly, the analysis revealed that knowledge and barriers were the most prominent factors influencing nurse compliance according to the model, as shown in Fig. 1.

## Discussion

To our knowledge, this is the first national and regional observational study to investigate critical care nurses’ compliance with CLABSI prevention guidelines and identify gaps in knowledge, attitudes, and perceived barriers. Furthermore, this study offers new insights into CLABSI prevention by highlighting the discrepancies between evidence-based recommendations and current practices among critical care nurses. The findings underscore the importance of knowledge, positive attitudes, and the need to overcome perceived barriers to promoting compliance with CLABSI prevention guidelines among critical care nurses. The overall mean score of Yemeni critical care nurses' compliance with CLABSI prevention guidelines barely reached an acceptable level (12.54 out of 20). Compliance with several critical measures was low. This may be linked to perceived barriers by nurses, according to the study's findings, such as staffing shortages, high workloads, lack of written protocols and in-service workshops/training programs in most hospitals, and unfamiliarity with guidelines. This is further compounded by the current state of Yemen's healthcare system, which is characterized by poor working conditions and overcrowded hospitals.

Critical care nurses in this study demonstrated a concerning gap in their knowledge (5.38 out of 10) regarding CLABSI prevention guidelines. These gaps put patients at increase the risk of developing CLABSIs. Some awareness existed in knowledge, such as recommendations for replacing CVCs routinely (64.9%), replacing the administration set when neither lipid emulsions nor blood products were administered (64.7%), and applying an antibiotic ointment at the insertion site of the CVC (60.2%). However, knowledge gaps were identified in recommendations regarding covering insertion sites, using CVC coated or impregnated with an antiseptic agent, and disinfecting the catheter insertion site. These findings contrast with a Malaysian study by Sham et al. (2023). This study found a lower mean score for knowledge (5.85) and lower scores in the same specific aspects (ranging from 19.7% to 23.2%), but also reported a higher percentage in other areas [13]. A comparison with studies by Chi et al. (2020) (16.29%, 10.90%, 21.32%, and 28.38%, respectively) also revealed a lower percentage [14]. Low knowledge was observed in items related to the time to change the IV set, using catheters impregnated with disinfectants, changing, cleaning, drying, and healthy dressing, and the best solution to disinfect the catheter entry site [11]. Incorrect answers were often provided for changes in administration sets, changes in pressure transducers and tubing, changes in transparent catheter dressings, and use of > 0.5% chlorhexidine [10]. Studies across China [14], 25 European countries [8], Jordan[15], Egypt [16], and Greece [17] point to a lack of knowledge regarding CLABSI prevention among ICU nurses. These findings are consistent with those of the present study. Conversely, studies from Poland, Italy, and Belgium reported higher knowledge levels [8, 18, 19]. Azlan et al. (2021) found that ICU nurses exhibit a high level of knowledge, attitudes, and practices regarding CLABSI prevention. The study also revealed a positive association between these three factors [20]. These discrepancies in studies on nurses' knowledge arise from several factors, including regional variations, hospital policies, tools used, national regulations, and nurse populations.

The study results revealed CLABSI prevention compliance gaps (12.54 out of 20). Critical care nurses exhibited low compliance with specific elements, such as using sterile gloves and maintaining clean dressings. Most critically, they frequently omitted two crucial steps: the dressing was maintained clean and dry, the catheter was flushed with normal saline, and the port was swabbed with antiseptic solution. This discrepancy in adherence can be attributed to two potential causes. One possibility is a knowledge gap regarding the critical nature of these steps. Moreover, nurses reported several contributing factors. These barriers to compliance include high workloads, staffing shortages, and limited access to necessary equipment. Deficiencies in compliance and reporting factors also contribute to an increased risk of CLABSIs. A similar result has been observed in nurses with handwashing, wearing sterile gloves, daily assessment of catheter insertion, and checking dressing change dates [12]. A low mean score for nurses’ practice has been reported by 4.19 in Malaysia, [13]. While (43%) of the nurses always followed the maximum barrier precautions, a concerning number (14%) never used chlorhexidine gluconate 2%. Additionally, only 40% of catheters were removed when no longer needed, and a third (33%) routinely changed catheters, even if there was no potential risk of infection [14]. A similar barrier factor was reported among Iranian nurses, with high workload, shortage of necessary equipment, and lack of workshops being the most common barriers [11]. A shortage of nurses, unfamiliarity with guidelines, and excessive workload are the main barriers for ICU nurses in China [10].

Nurses with higher education and access to prevention training and protocols exhibited higher knowledge, compliance, and positive attitudes toward CLABSI prevention. Conversely, they also reported fewer perceived barriers. This finding suggests that advanced education, experience, training, and clear policies play a role in enhancing nurses’ understanding, compliance, attitudes, and ability to overcome barriers to the implementation of CLABSI prevention guidelines in CVC care. This is supported by the finding that training, written policies, and experience contribute to an increase in knowledge, practice, and positive attitudes toward CLABSI prevention [9]. Nurses’ experiences were associated with their knowledge and practices. No association was found between nurses’ characteristics and their attitudes [13]. Age, sex, educational level, experience, and previous workshops associated with knowledge [11]. Experience and educational level are associated with knowledge [10]. Nurses’ knowledge differed according to age, ICU experience, and nurse-to-patient ratio. Moreover, nurse-to-patient ratio and hospital were associated with nurses’ compliance, whereas educational level and training were not associated with knowledge [21].

Interestingly, nurses working in teaching hospitals displayed higher levels of knowledge, compliance, and attitude. However, these barriers remain unchanged. This suggests that, while a teaching environment fosters positive learning and practice, it may not directly address perceived barriers in implementing CLABSI prevention. Our findings align with the study by Matlab (2022), which demonstrates an association between the type of hospital and nurses' compliance with implementing CLABSI prevention guidelines [21].

Nurses caring for one patient in the ICU demonstrated higher knowledge and compliance, and lower barrier scores than those caring for three patients. This finding suggests that a lower patient-to-nurse ratio facilitates more focused care, potentially enhancing knowledge acquisition, adherence to protocols, and overcoming barriers to implementing CLABSI preventive measures. Consequently, this approach may reduce the incidence of CLABSI, thereby highlighting the potential benefits of lower nurse-to-patient ratios. In addition, nurses facing higher barriers had lower compliance rates. Our multilayer neural network analysis identified knowledge as the most significant factor that influences nurse compliance. This suggests that the model prioritizes knowledge when evaluating and improving factors affecting nurses' compliance. This is consistent with a study in Saudi Arabia, which stated that previous education and nurses working in a 1:1 nurse-patient ratio environment were more likely to comply with CLABSI prevention guidelines than those working in a 1:2 ratio environment [12]. Moreover, the nurse-to-patient ratio is associated with nurses’ knowledge and compliance [21]. A low nurse-to-patient ratio reduces the incidence of CLABSIs [22]. Yemen's constant political violence, instability, and worsening economic situation are the root causes for everything. This has ultimately resulted in poor quality healthcare services and the daily stress and frustrations faced by the Yemeni people. These findings highlight the importance of education, patient care ratio, experience, availability of protocols, hospital type, attitudes, and barriers to influencing critical care nurses knowledge and compliance.

To effectively prevent hospital-acquired infections, especially CLABSI, it is essential to regularly assess the knowledge and compliance of critical care nurses and to address barriers. This evaluation can be effortlessly integrated into the existing competency assessments for critical care nurses. Moreover, ongoing monitoring of nurses' adherence to the latest evidence-based CVC care bundles can provide useful feedback for identifying areas where further education is needed. This feedback loop can inform the development and implementation of targeted educational interventions, such as workshops or training sessions, to improve the ability of critical care nurses to prevent CLABSI or other hospital-acquired infections.

## Limitations

The limitations of the present study include its cross-sectional design, which precludes the establishment of causal relationships between the variables. data limitations regarding the number of ICU nurses hindered the ability to calculate the optimal sample size. The use of a convenience sample leads to a potential for selection bias and presents challenges in generalizing study findings. To a certain extent, large sample size can help mitigate this bias. The investigation was limited to critical care nurses and did not include physicians. Observing nurses' compliance might introduce a Hawthorne effect, whereby nurses alter their behavior in response to being observed. Moreover, the results are specific to critical care nurses in Sana'a City, and may not be generalizable to other regions. Nonetheless, one notable strength of this study is the inclusion of various ICUs and hospitals, which provides an accurate representation of the geographic region under investigation.

## Conclusion

Critical care nurses displayed limited knowledge of CLABSI prevention guidelines, with compliance rates barely reaching an acceptable level. Despite encountering more perceived barriers, nurses maintained positive attitudes towards CLABSI prevention. Moreover, nurses with greater knowledge and more positive attitudes exhibited higher levels of compliance. However, perceived barriers were negatively associated with knowledge and compliance. Remarkably, multilayer neural network analysis revealed that knowledge and perceived barriers were the strongest predictors of nurses' compliance. This study provides invaluable insights into the factors that influence the implementation of CLABSI prevention guidelines among critical care nurses. This emphasizes the need for comprehensive strategies to simultaneously address these factors. Compliance rates can be significantly enhanced by improving knowledge, fostering positive attitudes, and finding solutions to daily barriers.

## Relevance to clinical practice

A deficit in critical care nurses' knowledge, compliance with, and the presence of significant barriers hindering the implementation of CLABSI prevention guidelines poses a potential threat to patient safety. These findings underscore the necessity for healthcare managers to prioritize initiatives to improve nurses' training and knowledge of CLABSI prevention. Additionally, addressing and mitigating the identified barriers is crucial for optimizing the implementation of CLABSI guidelines.

### Supplementary Information

Supplementary Material 1.

## Data Availability

Data available on request from the authors.

## References

[1] Sellamuthu R, Nair S, Chandrasekar J, Kesavan S, Shivam V. Risk Factors of Central Line-Associated Bloodstream Infection (CLABSI): A Prospective Study From a Paediatric Intensive Care Unit in South India. Cureus. 2023;15(8): e43349. 37700998 10.7759/cureus.43349 PMC10493200

[2] Lafuente Cabrero E, Terradas Robledo R, Civit Cuñado A, García Sardelli D, Hidalgo López C, Giro Formatger D, Lacueva Perez L, Esquinas López C, Tortosa Moreno A. Risk factors of catheter-associated bloodstream infection: systematic review and meta-analysis. PLoS ONE. 2023;18(3): e0282290. 36952393 10.1371/journal.pone.0282290 PMC10035840

[3] Cooper M. Improving Nurses' Knowledge of Central Line-Associated Bloodstream Infection (Publication Number 6190). Washington: Walden University; 2019. https://scholarworks.waldenu.edu/dissertations/6190/.

[4] Haddadin Y, Annamaraju P, Regunath H. Central Line–Associated Blood Stream Infections. 2022. 28613641

[5] Control CfD. Prevention: Central line-associated bloodstream infections: resources for patients and healthcare providers. 2011.

[6] Jun J, Kovner CT, Stimpfel AW. Barriers and facilitators of nurses’ use of clinical practice guidelines: an integrative review. Int J Nurs Stud. 2016;60:54–68. 27297368 10.1016/j.ijnurstu.2016.03.006

[7] Austin PC, Steyerberg EW. The number of subjects per variable required in linear regression analyses. J Clin Epidemiol. 2015;68(6):627–36. 25704724 10.1016/j.jclinepi.2014.12.014

[8] Labeau S, Vereecke A, Vandijck D, Claes B, Blot S. Critical care nurses’ knowledge of evidence-based guidelines for preventing infections associated with central venous catheters: an evaluation questionnaire. Am J Crit Care. 2008;17(1):65–71. 18158392 10.4037/ajcc2008.17.1.65

[9] Bianco A, Coscarelli P, Nobile CG, Pileggi C, Pavia M. The reduction of risk in central line-associated bloodstream infections: knowledge, attitudes, and evidence-based practices in health care workers. Am J Infect Control. 2013;41(2):107–12. 22980513 10.1016/j.ajic.2012.02.038

[10] Chen S, Yao J, Chen J, Liu L, Miu A, Jiang Y, Zhu J, Tang S, Chen Y. Knowledge of “Guidelines for the prevention of intravascular catheter-related infections (2011)”: A survey of intensive care unit nursing staffs in China. International Journal of Nursing Sciences. 2015;2(4):383–8. 10.1016/j.ijnss.2015.10.002

[11] Badparva B, Ghanbari A, Karkhah S, Osuji J, Kazemnejad Leyli E, Jafaraghaee F. Prevention of central line-associated bloodstream infections: ICU nurses’ knowledge and barriers. Nurs Crit Care. 2023;28(3):419–26. 35118750 10.1111/nicc.12757

[12] Aloush SM, Alsaraireh FA. Nurses’ compliance with central line associated blood stream infection prevention guidelines. Saudi Med J. 2018;39(3):273. 29543306 10.15537/smj.2018.3.21497 PMC5893917

[13] Sham F, Sulaiman NH, Seman A, Shohor NA, Mun CY. Intensive care nurses'knowledge, practice and attitude in prevention of central line-associated bloodstream infection (CLABSI). J Health Transl Med (JUMMEC). 2023;(2):102–10.

[14] Chi X, Guo J, Niu X, He R, Wu L, Xu H. Prevention of central line-associated bloodstream infections: a survey of ICU nurses’ knowledge and practice in China. Antimicrob Resist Infect Control. 2020;9:1–9. 33198796 10.1186/s13756-020-00833-3 PMC7667726

[15] Al Qadire M, Hani AM. Nurses’ and physicians’ knowledge of guidelines for preventing catheter-related blood stream infections. Nurs Crit Care. 2022;27(4):594–601. 33325063 10.1111/nicc.12577

[16] Sobeih H, Nabil Abd-Elsalam S, Ramadan S. Infection Control Measures for Patient with Central Line: Nurses’ Performance. Egyptian Journal of Health Care. 2018;9(2):227–37. 10.21608/ejhc.2018.20267

[17] Koutzavekiaris I, Vouloumanou EK, Gourni M, Rafailidis PI, Michalopoulos A, Falagas ME. Knowledge and practices regarding prevention of infections associated with central venous catheters: a survey of intensive care unit medical and nursing staff. Am J Infect Control. 2011;39(7):542–7. 21496955 10.1016/j.ajic.2010.11.003

[18] Esposito MR, Guillari A, Angelillo IF. Knowledge, attitudes, and practice on the prevention of central line-associated bloodstream infections among nurses in oncological care: A cross-sectional study in an area of southern Italy. PLoS ONE. 2017;12(6): e0180473. 28665993 10.1371/journal.pone.0180473 PMC5493401

[19] Dyk D, Matusiak A, Cudak E, Gutysz-Wojnicka A, Mędrzycka-Dąbrowska W. Assessment of knowledge on the prevention of central-line-associated bloodstream infections among intensive care nurses in poland—a prospective multicentre study. Int J Environ Res Public Health. 2021;18(23):12672. 34886399 10.3390/ijerph182312672 PMC8657192

[20] Azlan N, Aung K. Knowledge, Attitude and Practices of ICU Nurses on Catheter Related Bloodstream Infection (CRBSI). Int J Crit Care Emerg Med. 2021;7:125.

[21] Matlab AA, Al-Hussami MO, Alkaid Albqoor M. Knowledge and compliance to prevention of central line-associated blood stream infections among registered nurses in Jordan. J Infect Prev. 2022;23(4):133–41. 37256157 10.1177/17571774211066778 PMC10226055

[22] Ball M, Singh A. Care of a Central Line. StatPearls Publishing LLC; 2023. https://www.ncbi.nlm.nih.gov/books/NBK564398/. Accessed 25 Mar 2024. 33232068

