# Supplementary material for: Mitigating risks in central line-associated bloodstream infection: a comprehensive insight into critical care nurses’ knowledge, attitudes, barriers, and compliance
Source: BMC Nurs. 2024 Jul 20;23:497. doi: 10.1186/s12912-024-02168-5 (PMC11265045; doi:10.1186/s12912-024-02168-5)
Supplement: Supplementary file 1 — Supplementary Material 1. [file 12912_2024_2168_MOESM1_ESM.docx]

**Appendix**

Critical are nurses’ knowledge of CLABSI prevention guideline.

| **Knowledge Items** | **Correct answer** | **Incorrect answer** |
| --- | --- | --- |
| It is recommended to replace central venous catheters (CVCs) routinely | 305(64.9) | 165(35.1) |
| It is recommended to replace CVCs over a guidewire | 251(53.4) | 219(46.6) |
| It is recommended to replace pressure transducers and tubing routinely | 235(50) | 235(50) |
| In settings with a high rate of catheter-related infections it is recommended to use a CVC coated or impregnated with an antiseptic agent | 206(43.8) | 264(56.2) |
| It is recommended to change the dressing on the catheter insertion site | 246(52.3) | 224(47.7) |
| It is recommended to cover up the catheter insertion site with | 194(41.3) | 276(58.7) |
| It is recommended to disinfect the catheter insertion site with | 208(44.3) | 262(55.7) |
| It is recommended to apply an antibiotic ointment at the insertion site of a CVC | 283(60.2) | 187(39.8) |
| When lipid emulsions are administered through a CVC it is recommended to replace the administration set | 297(63.2) | 173(36.8) |
| When neither lipid emulsions nor blood products are administered through a CVC it is recommended to replace the administration set | 304(64.7) | 166(35.3) |

Critical are nurses’ compliance of CLABSI prevention guideline.

| **Compliance Items** | **Not done** | **Done incompletely** | **Done completely** |
| --- | --- | --- | --- |
| Daily assessment of the catheter insertion site | 17(3.6) | 153(32.6) | 300(63.8) |
| Assessment of the date is made for dressing | 111(23.6) | 132(28.1) | 227(48.3) |
| Dressing is maintained clean and dry | 213(45.3) | 94(20) | 163(34.7) |
| Hand washing | 52(11.1) | 95(20.2) | 323(68.7) |
| Sterile Gloves | 134(28.5) | 195(41.5) | 141(30) |
| Swap port with antiseptic | 134(28.5) | 107(22.8) | 229(48.7) |
| Flush with Normal Saline 0.9% | 182(38.7) | 115(24.5) | 173(36.8) |
| Change intravenous sets | 121(25.7) | 117(24.9) | 232(49.4) |
| Cover all lumens when not in use | 43(9.1) | 162(34.5) | 265(56.4) |
| Use minimum number of lumen unless in need | 124(26.4) | 75(16) | 271(57.7) |
